# The Role of Extracellular Vesicles (EVs) in the Epigenetic Regulation of Bone Metabolism and Osteoporosis

**DOI:** 10.3390/ijms21228682

**Published:** 2020-11-17

**Authors:** Maurizio Muraca, Alfredo Cappariello

**Affiliations:** 1 Department of Women’s and Children’s Health, University of Padova, 35122 Padova, Italy; maurizio.muraca@aopd.veneto.it; 2 Research Laboratories, Department of Onco-hematology, Pediatric Hospital Bambino Gesù, 00146 Rome, Italy

**Keywords:** epigenetics, long non-coding RNA, microRNA, osteoblast, osteoclast, osteocyte, osteoporosis, bone metabolism

## Abstract

Extracellular vesicles (EVs) are complex phospholipidic structures actively released by cells. EVs are recognized as powerful means of intercellular communication since they contain many signaling molecules (including lipids, proteins, and nucleic acids). In parallel, changes in epigenetic processes can lead to changes in gene function and finally lead to disease onset and progression. Recent breakthroughs have revealed the complex roles of non-coding RNAs (microRNAs (miRNAs) and long non-coding RNAs (lncRNAs)) in epigenetic regulation. Moreover, a substantial body of evidence demonstrates that non-coding RNAs can be shuttled among the cells and tissues via EVs, allowing non-coding RNAs to reach distant cells and exert systemic effects. Resident bone cells, including osteoclasts, osteoblasts, osteocytes, and endothelial cells, are tightly regulated by non-coding RNAs, and many of them can be exported from the cells to neighboring ones through EVs, triggering pathological conditions. For these reasons, researchers have also started to exploit EVs as a theranostic tool to address osteoporosis. In this review, we summarize some recent findings regarding the EVs’ involvement in the fine regulation of non-coding RNAs in the context of bone metabolism and osteoporosis.

## 1. Introduction

Osteoporosis is defined by the World Health Organization (WHO) as a progressive systemic skeletal disorder with low bone density and deterioration of bone architecture leading to an increased risk of bone fragility and fracture [1,2]. Currently, osteoporosis is a major public health problem. Although osteoporosis is not a severe disease per se, the healthcare cost is massive, especially for the management of the fractures resulting from low bone mass, associated with a decreased quality of life and lifespan in aging people. About 200 million people (1 in 8 women after 55 years of age and 1 in 4 men after 65 years of age) suffer from osteoporosis worldwide with an associated incidence of 9 million fractures [3,4]. The problem is growing in an aging population. In women, the increase in the bone remodeling rate in both cortical and cancellous bone, combined with a negative remodeling balance, results in bone loss and collapse of bone microarchitecture. Indeed, cortical bone peculiarly shows an increase in cortical porosity and a reduction in cortical thickness, while cancellous bone is mainly affected by thinning and loss of trabeculae [5,6,7]. In men, a reduction in bone formation and bone turnover occurs during aging. Changes in matrix and mineral composition of bone can also contribute to increased bone fragility [8]. It has been shown that osteoporosis has genetic and molecular heterogeneity like many other common complex diseases [3]. The era of genome-wide association studies (GWAS) clarifies the horizon of the genetic contribution to osteoporosis. Many GWAS have been performed interrogating genetic association with bone mineral density (BMD), implicating more than 90 candidate genes for osteoporosis [9]. In this context, non-coding RNAs were identified as playing a crucial role as a genomic regulatory system in several biological processes of bone metabolism [10,11]. Interestingly, some GWAS reported association between polymorphisms of ncRNAs and BMD. Zeng et al. conducted a meta-analysis among individuals of seven independent studies for BMD, reporting that MEF2C antisense RNA 1 (MEF2C-AS1) was significantly associated with BMD of femoral neck, while LOC100506136 showed significant association with BMD of hip [12]. Similarly, Zhang et al. constructed a comprehensive mRNA–lncRNA–miRNA competing endogenous RNA (ceRNA) regulatory network to predict the risk of osteoporosis development by analyzing public Gene Expression Omnibus (GEO) microarray profile data [13]. In the next paragraphs, we provide information about the mechanisms and the role of non-coding RNAs in the regulation of gene expression and their link with extracellular vesicles (EVs) in the onset of osteoporosis.

### 1.1. Cellular Basis of Bone Metabolism and Osteoporosis

Osteoporosis occurs when the bone turnover is perturbed and bone mass decreases [1]. Bone mass is maintained by the synchronized and fine-tuned crosstalk between bone building cells, osteoblasts, and bone-resorbing cells, i.e., osteoclasts. On the one hand, osteoblasts, deriving from mesenchymal precursors, are responsible for the deposition of both the organic (mainly collagen type I) and inorganic (hydroxyapatite Ca_10_(PO_4_)_6_(OH)_2_) constituents of the extracellular matrix [14]. On the other hand, osteoclasts, multinucleated giant cells arising from the fusion of mononuclear hematopoietic precursors of the monocytic/macrophagic lineage, degrade the extracellular matrix through a very specialized and unique structure, the resorption lacuna, which allows the hydrolysis of hydroxyapatite through the acidification of extracellular space and the subsequent exposition of collagen fibrils, degraded by metalloproteases and cathepsin K [15,16]. The activity of these cells is strictly coordinated in time and space, and the interdependence of these cells is such that they are grouped into one histological and functional unit, called the bone remodeling unit [17,18]. However, these two cell types are not the only players on the stage [18]. In fact, the most abundant (>90%) cells in the bone are the osteocytes, deriving from the embedding in the bone matrix of mature osteoblasts [19]. Osteocytes, previously considered static bystanders entrapped into the bone, are now known to play a crucial role in sensing mechanical loading and regulating calcium and phosphate homeostasis [20]. These signals are integrated and reported to the bone remodeling unit, since osteocytes create a tight web across the skeleton with the projection of 40–100 dendritic extensions per cell, which spread through the bone matrix in channels called canaliculi, forming connections to neighboring osteocytes, osteoblasts, osteoclasts, and blood vessels. Many molecular mechanisms concur to synchronize the osteoblast/osteoclast/osteocyte activity. The main one is the receptor activator of NF-κB ligand, RANKL [15,21]. This cytokine, belonging to the tumor necrosis factor (TNF) superfamily members, is expressed on the surface of osteoblasts and osteocytes. The binding of RANKL to its receptor RANK on the membrane of osteoclast precursors and mature osteoclasts triggers the osteoclast formation and sustains the osteoclast function. This ligand–receptor binding can be prevented by osteoprotegerin (OPG), a soluble decoy of RANKL, produced by osteoblasts and osteocytes. In addition, osteocytes influence in a paracrine manner both osteoblast and osteoclast activity, i.e., osteocytes regulate bone formation by secreting modulators of the wingless-type mouse mammary tumour virus [MMTV] integration site members (Wnt) signaling pathway such as activators (nitric oxide and ATP) and inhibitors (sclerostin (SOST) and dickkopf-related protein 1 (DKK1)). Similarly, osteocytes regulate bone resorption, other than by RANKL and OPG, by releasing nitric oxide, an inhibitor of osteoclast formation and activity [22]. The amounts of bone resorbed and formed in each bone remodeling unit are important determinants of bone mass. This continuous bone remodeling process is required to replace old bone tissue and to repair bone micro-cracks [17]. In adults, it has been estimated that 10% of bone is replaced every year and that this is crucial for the maintenance of a healthy skeleton [17].

Many determinants, both genetic and environmental, contribute to set bone mass and guarantee bone health. Steroid hormones exert a crucial role in reaching (during puberty) and maintaining (at adulthood) the peak of bone mass and the correct BMD level among both women and men [23]. In fact, the decline of estrogen and testosterone during menopause and andropause is one of the major risk factors and drivers of osteoporosis [23,24]. Hormonal withdrawal also contributes to mineral disturbances. Indeed, intestinal calcium absorption is decreased and urinary calcium excretion is increased, thus leading to a negative calcium balance and secondary hyperparathyroidism, exacerbating osteoclast activity [25,26]. Physical exercise and muscle fitness have a dramatic impact on bone health. In fact, the mechanical force produced during muscular activity is sensed by osteocytes and converted into bone deposition, whereas disuse and/or muscle atrophy result in osteoporosis [27,28]. Moreover, muscle secretes a set of molecules, known as myokines, directly affecting bone metabolism, such as irisin, myostatin, and insulin-like growth factor-1 [29].

### 1.2. Diagnosis and Biomarkers of Osteoporosis

The standard clinical technique for diagnosis of osteoporosis is the dual-energy x-ray absorptiometry (DXA) of the spine or hip [21]. DXA reports the T-score, a quantified measure of BMD. A T-score between −1 and −2.4 standard deviations compared to the mean score of a healthy young woman defines an osteopenic condition, whereas a T-score greater than or equal to −2.5 standard deviations is considered diagnostic for osteoporosis [22]. BMD values are also routinely used to monitor fracture risk and bone health. However, BMD has several limitations, i.e., it is not effective and sensitive as a surveillance tool, especially in the case of minimal changes during a pharmacological treatment, and the T-score is not in many cases a reliable predictor of fragility fractures. Due to these limitations, the management of osteoporosis in most cases mainly relies on biomarkers [30,31]. These are mostly molecules released by bone cells during bone remodeling, measurable in urine or serum, and are indicative of the rate of bone turnover. Bone biomarkers can be classified into markers of bone formation or bone resorption [32]. The first class groups total alkaline phosphatase (total ALP), bone-specific alkaline phosphatase (B-ALP), procollagen type 1 *N*-terminal propeptide (P1NP), osteocalcin (OC), and procollagen type 1 C-terminal propeptide (P1CP). The second class includes hydroxyproline (HYP), pyridinoline, tartrate-resistant acid phosphatase 5b (TRAP 5b), deoxypyridinoline (DPD), carboxy-terminal cross-linked telopeptide of type 1 collagen (CTX-1), and amino-terminal cross-linked telopeptide of type 1 collagen (NTX-1).

### 1.3. Drugs and Therapeutic Strategies for Osteoporosis Management

Pharmacological opportunities for osteoporosis aim to restore the steady activity of the bone remodeling unit. Bone anti-resorptive and bone anabolic agents are available in clinical management [32].

#### 1.3.1. Anti-Resorptive Agents

Anti-resorptive drugs are the most commonly used pharmacological tool to avoid osteoporosis progression. These drugs abrogate the exacerbated osteoclast activity, preserving the existing bone mass and structure. Bisphosphonates (alendronate, risedronate, and zoledronic acid) are the first-in-use drugs used to inhibit bone resorption and, to date, are still largely used in the clinical management of osteoporosis [30,33]. The newest agent denosumab, a fully human monoclonal antibody, binds to and inhibits RANKL, resulting in a marked but reversible inhibition of bone resorption.

#### 1.3.2. Anabolic Agents

Anabolic agents aim to improve osteoblast function and stimulate bone deposition and mineralization. Estrogen-replacing therapy prevents bone loss in postmenopausal women [34]. Use is limited in early menopause, no more than 10 years, because of concerns about cardiovascular safety [35]. Selective estrogen receptor modulators (SERMs, such as raloxifene and bazedoxifene) bind with high affinity to the estrogen receptor and have both agonist and antagonist properties depending upon the target tissue [36]. SERMs present the same procoagulant concerns of estrogen, but exert a beneficial effect to reduce breast cancer [37]. Parathyroid hormone (PTH) receptor agonists, e.g., teriparatide (recombinant fragment of human PTH consisting of the first 34 amino acids) and abaloparatide (analogue of PTH-related peptide, PTHrP) improve bone mass and reduce fracture through binding of the PTH receptor-1 on osteoblasts [38,39]. The newest FDA-approved anabolic agent is romosozumab, a monoclonal antibody that binds to SOST [40]. Due to the inhibition of Sost, romosozumab both increases bone formation and decreases bone resorption.

## 2. Epigenetic Regulation

Epigenetics is the study of inherited changes in phenotype or gene expression that are caused by mechanisms other than changes in the structural DNA sequence [41,42]. These modifications may persist through several cell divisions lasting for generations or can be acquired permanently. The most noteworthy epigenetic mechanisms include the following: (1) DNA methylation, through which the genome is locally regulated inserting a methyl group tag on DNA by DNA methyltransferases; (2) histone modifications, through which the nucleosome superstructure is regulated by reversibly modulating the DNA wrapping extent around histones by histone methyltransferases and histone acetyltransferases; and (3) processes mediated by the most recently discovered class of RNAs, the non-coding RNAs [43]. Although actively transcribed, these RNAs are not translated into proteins and can be generally divided into two categories based on size, namely short-chain non-coding RNAs (sncRNAs) and long non-coding RNA (lncRNAs) [43,44,45]. A better ncRNA classification relies on the structure or function. microRNAs (miRNAs), which are 19–24 nucleotides in length, usually recognize target mRNAs by complementarity to a 2–7 nucleotide long seed region in the 3′-UTR region, although the seed region can be also located at the 5′-UTR and CDS regions. Their biogenesis is dependent on Drosha, Dicer, and Argonautes (AGO) and, finally, activating RNA-induced silencing (RISC) complex [46,47]. The Piwi-interacting RNAs (piRNAs), are 26–30 nucleotide single-stranded RNAs, are Dicer independent, and interact with Piwi-domain-containing proteins [48]. The piRNA precursor transcripts undergo endonucleolytic cleavage in the cytoplasm and are complexed with the Aubergine (Aub) or Piwi chaperone complex for 3′ trimming and methylation, finalizing the primary biogenesis pathway. Natural small interfering RNAs (siRNAs) are 20 to 24 base pair double-stranded RNAs (dsRNAs) with phosphorylated 5′ ends and hydroxylated 3′ ends with two overhanging nucleotides [43,49]. Upon Dicer cleavage, a shorter double-strand duplex is formed ready to enter into the RISC. Small nuclear RNAs (snRNAs) and small nucleolar RNAs (snoRNAs), are approximately 150 nucleotide transcripts found within the nucleus of eukaryotic cells, involved in the processing of pre-messenger RNA, in the regulation of transcription factor, and in the maintaining of telomeres, or in ribosomal RNA modification and processing in the nucleolus [48]. Finally, the long non-coding RNAs can be classified as follows, according to their structural relationship with the adjacent protein-coding genes: (a) sense or antisense, lncRNAs are located and transcribed on the same or the opposite strand of the adjacent protein-coding genes; (b) convergent or divergent, lncRNAs have a convergent (divergent) orientation of transcription compared with that of the adjacent protein-coding genes; and (c) intergenic or intronic, lncRNAs are located between two protein-coding genes, or located in introns [45,50,51,52,53]. The most abundant isoform of lncRNAs are predicted to be circular RNAs (circRNAs) compared to a linear transcript. CirclncRNAs can be formed via canonic splicing (intronic circRNAs) or via backsplicing, joining the splice donor at the upstream acceptor site [54,55,56]. Another classification of lncRNAs is relative to their functions, as discussed below (see Section 2.2).

In recent years, a large number of studies have shown that non-coding RNAs play a significant role in epigenetic modifications at post-transcriptional or promoter levels, since they can regulate gene expression both at the level of the gene itself and at the level of the chromosome to control cell differentiation [57,58,59].

### 2.1. miRNAs

**Figure 1:**
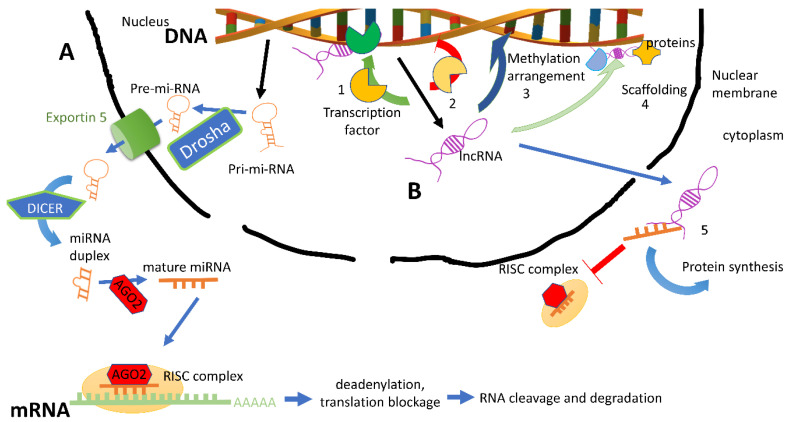
Cartoon summarizing the actions of microRNAs (miRNAs) and long non-coding RNAs (lncRNAs) in the cell. The processes of biogenesis and sorting in the cellular compartment as well as the interaction with molecular partners are highlighted. (**A**) Drosha, a nuclear endonuclease, converts the hairpin-loop RNA primary transcript into a shorter stem-loop structured pre-miRNA. After nuclear export by exportin 5, Dicer cleaves the pre-miRNA stem-loop into an RNA fragment with a two-nucleotide 3′ overhang at each end. Thereafter, the mature miRNA is loaded into the RISC complex by AGO proteins (mainly AGO2). Finally, the target mRNA is sorted and processed for cleavage. (**B**) LncRNA can work in the nucleus or can be exported into the cytoplasm. In the nucleus, lncRNA can act as (1) signaling, synergistically cooperating with a transcription factor to finely regulate in time and space the gene expression; (2) decoy, undermining a transcription factor; (3) guide, recruiting chromatin-modifying enzymes on a target gene; or (4) scaffold, favoring the formation of a complex or a spatial proximity of two or more proteins. When lncRNA is exported into the cytoplasm, it can sequester a miRNA, acting as a sponge (5), avoiding the assembly of RISC complex and favoring the translation of the mRNA into a protein.

miRNAs are single-stranded RNAs of approximately 19–24 nucleotides, of which 50% are located in non-coding chromosomal regions [60]. During biogenesis (Figure 1), a primordial hairpin-shaped double-stranded intermediate is maturated by Drosha and DICER enzymes [61]. Finally, the mature miRNA is complexed with the loading proteins AGO, being able to target a specific messenger RNA through the assembly with RISC [46]. Almost 1800 putative miRNAs have been identified in the human genome, and the number of miRNAs is still increasing rapidly due to the progress in sequencing and bioinformatic technologies [62]. Interestingly, a single miRNA can target up to hundreds of different genes [63]. Histone methyltransferases, methyl CpG-binding proteins, chromatin domain proteins, and histone deacetylases are assumed to be putative targets for miRNAs [63]. For example, histone deacetylase 4 (HDAC4) has been demonstrated to be a specific target for miR-140 in mouse embryonic cartilage tissue [64]. miRNAs (i.e., the miR-29 family) can also regulate DNA methylases, affecting DNA methyltransferases 3A and 3B [65].

miRNA dysfunctions have been associated with different pathologies, including osteoporosis. Three miRNAs (miR-21, miR-133a, and miR-146a) have been found differentially expressed in the plasma of 120 Chinese postmenopausal women according to their T-scores [20]. In the serum of osteoporotic patients, Seelinger and colleagues identified five overexpressed miRNAs (miR-100, miR-122, miR-124a, miR-125b and miR-148a) associated with a high fracture risk [66]. Along the same lines, another group confirmed the differential expression of five miRNAs (miR-10a/b, miR-133b, miR-21-5p, and miR-22-3p) in the serum of postmenopausal women suffering from recent femoral-neck fractures [67].

### 2.2. Long Non-Coding RNAs (lncRNAs)

lncRNAs are a newly discovered class of non-coding regulatory RNAs generally containing more than 200 nucleotides. They are located in the nucleus or cytoplasm, and rarely encode proteins [68]. lncRNAs can be generated by the following five main mechanisms: (a) disruption of the translational reading frame of a gene, (b) chromosomal reorganization, (c) retrotransposition of a non-coding gene, (d) partial tandem duplication of a non-coding gene, and (e) insertion of a transposable element into a gene producing a functional non-coding gene [45]. lncRNAs can behave as signal, favoring gene expression; decoy, suppressing gene expression; guide, promoting chromatin modification; or scaffold, acting on a chromatin structure [69]. Furthermore, lncRNAs can work as a sponge for miRNAs, since they can harbor recognition sites for miRNAs. Due to the complementarity of the sequences, lncRNAs can sequester miRNA and prevent it from repressing its mRNA target, resulting in increased expression of protein [70]. In this view, lncRNA *H19* has been reported to enhance osteoblast proliferation by sequestering miR-141 and miR-22, negative regulators of osteogenesis and the Wnt/β-catenin pathway [71]. Similarly, lncRNA metastasis-associated lung adenocarcinoma transcript 1 (MALAT 1) acted as miR-1 sponge to inhibit its suppressive transcription effect on Connexin43 [72]. For these reasons, these lncRNAs were defined as competing endogenous RNAs (ceRNAs) (Figure 1). lncRNAs have been associated with the development of osteoporosis, and some authors proposed their use as a diagnostic and therapeutic tool for osteoporosis [73,74,75,76,77,78]. For example, X-inactive-specific transcript (Xist) RNA has been described to inhibit osteogenic differentiation bone marrow mesenchymal stem cell [79]. Interestingly, Xist was associated with the inhibition of the osteogenic potential of bone marrow mesenchymal stem cells, suppressing protein expression levels of ALP, OC, and runt-related transcription factor 2 (RUNX2) [79]. Accordingly, Xist was highly expressed in patients affected with osteoporosis [79]. Along the same lines, lncRNA ZBTB40-IT1 has been revealed as a regulator of bone metabolism, since it is able to suppress osteogenesis and promote osteoclastogenesis by regulating the expression of WNT4, RUNX2, Osterix (OSX), ALP, collagen type I alpha 1 chain (COL1A1), OPG, and RANKL in osteoblastic-like cells [80]. Similarly, low expression of lncRNA H19 has been detected in disuse osteoporosis, accompanied by the inhibition of osteogenesis and impaired trabecular bone growth [81].

## 3. Extracellular Vesicles

**Figure 2:**
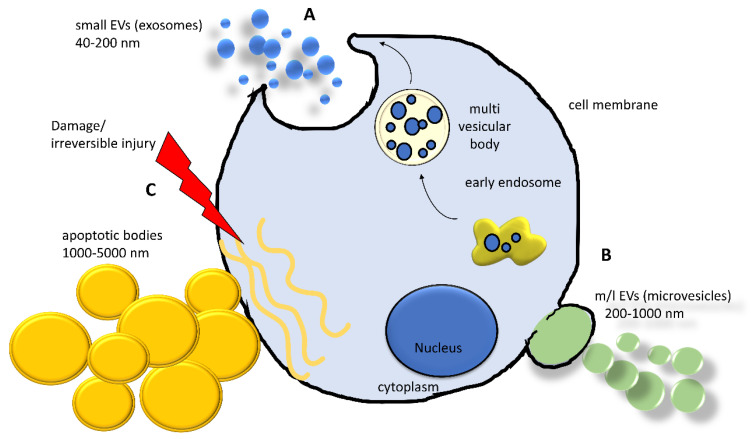
Schematization of the biogenesis and formation of the main classes of extracellular vesicles (EVs) released from a cell. (**A**) Early endosomes involved in the sorting and recycling of canonical intracellular vesicles can generate a multivesicular body (MVB), entrapping vesicles. An MVB can fuse with plasma membrane releasing its content in extracellular space. (**B**) Plasma membrane can undergo a complex remodeling by sophisticated molecular machinery, generating a bud from which a microvesicle is formed. (**C**) When a cell experiences a severe injury triggering irreversible damage, the cell activates the apoptotic pathway implying the organized dismantling of the cytoplasm. This process induces the release of the apoptotic bodies. sEVs = small extracellular vesicles; m/lEVs = medium/large extracellular vesicles.

Currently, the intricate scenario of cell-to-cell communication is further complicated by the recognition of the pivotal role of EVs. EVs are complex phospholipidic cell-derived structures actively released from cells in the environment and able to shuttle biological information across cells and tissues. Although the involvement of EVs in virtually all biological processes (i.e., embryogenesis, neuronal plasticity, immune response) was demonstrated in the last decade, the existence of EVs was reported by Wolf in 1967, associated with platelets and coagulation [82], and they were subsequently observed in reticulocyte differentiation, acting as a cellular garbage system [83]. Since then, extensive research activity in the field has led to a better—although largely incomplete—understanding of their biological properties and has laid the foundation for their use as diagnostic and therapeutic tools [84]. Extracellular vesicles are classified into the following three main classes, accordingly to Minimum Information for Studies of EVs (MISEV) guidelines: small extracellular vesicles (sEVs, diameter < 200 nm, also known as exosomes), medium/large extracellular vesicles (m/lEVs, diameter >200 nm up to 1000 nm, also known as microvesicles), and apoptotic bodies (>1000 nm up to 5000 nm) [85]. Apart from the size, the three classes of EVs differ in their biogenesis [86] (Figure 2). Small EVs derive from the release of a larger structure, called a multivesicular body (MVB), a component of the endocytic pathway that sorts sEVs in its lumen, finally releasing them by the fusion with the plasma membrane. This mechanism involves multiple protein partners, such as Ras-related proteins in brain (RAB), endosomal sorting complex required for transport (ESCTR) components, and proteins in the ceramide/sphingomyelinase pathway [86]. Medium/large shedding EVs, or microvesicles, bud from the cell surface by sprouting and scission of the membrane. ADP-ribosylation factor 6 (ARF6) activates the phospholipase D (PLD), resulting in a phospholipidic reorganization and thereby relocating phosphatidylserines toward the outer side of the membrane. Finally, extracellular signal-regulated kinase (ERK) is recruited to the plasma membrane and activates through phosphorylation the myosin light-chain kinase (MLCK), resulting in invagination of plasma membrane and release of EVs [86]. Apoptotic bodies arise from the cleavage of the cell during apoptosis, a way to neatly package the cell components in the surroundings and exert many biological effects [87,88].

Interestingly, all the resident bone cells, i.e., osteoblasts, monocytes/macrophages, osteoclasts, osteocytes, adipocytes, and endothelial cells, have been demonstrated to release EVs or respond to EVs both in physiological and pathological conditions [89,90,91,92,93,94,95,96]. EVs have been identified to shuttle molecules coming from the molecular legacy of the donor cells, exerting a specific effect according to the metabolic status of parental cells [97]. A multitude of studies defined the involvement of EVs in transferring genetic materials in many systems. For example, monocyte-derived EVs shuttle miR-155 to the endothelium, increasing endothelial cell migration [98]. Lv et al. demonstrated that vesicular tubular epithelial cells communicate by EVs shuttling miR-19b-3p to macrophages, leading to M1 macrophage switching [99]. Multiple myeloma cells under hypoxic conditions release miR-135b enriched sEVs able to decrease in target endothelial cells the factor-inhibiting hypoxia-inducible factor 1 (FIH-1), thereby increasing angiogenesis [100]. For a more detailed review on this aspect, see O’Brien et al. [101].

## 4. Control of Bone Metabolism by Means of EVs

Communication by means of EVs is a crucial mechanism involved in bone metabolism and intercellular crosstalk [14]. Some evidence in the late 1960s prompted researchers to postulate a role played by EVs in early mineral nucleation during cartilage mineralization [102,103]. More recently, Davies et al. demonstrated that mineralizing osteoblasts release EVs enriched in annexin 2, making the EV membrane able to complex octacalcium phosphate and other ions. This complex showed the intrinsic ability of triggering mineralization in an acellular context [104]. Furthermore, EVs are strictly related to cell-to-cell communication. Osteoclast differentiation and survival required the action of the irreplaceable cytokine receptor activator of NF-κB ligand (RANKL) [105,106,107]. Different groups described the release from osteoblast-like cells of EVs enriched in RANKL, directly supporting osteoclastogenesis [89,108]. In contrast, Ikebuchi et al. described that the mature osteoclasts produce EVs shuttling RANK [91]. Once bound to RANKL expressed on the osteoblast membrane, EVs trigger the reverse signaling pathway, inducing osteoblast maturation and bone deposition. Weilner and colleagues showed that endothelial cells produce EVs containing galectin-3, able to induce osteogenic differentiation on MSCs [109]. Adipocytes transfer via EVs adipocyte-specific transcripts such as *adiponectin, resistin*, and *peroxisome proliferator-activated receptor gamma 2* (*Pparγ2*) into macrophages [110] or *leptin*, *tumor necrosis factor alpha* (*Tnfα*), and *fibroblast growth factor alpha* (*Fgfα*) into endothelial cells, thereby inducing angiogenesis [111]. Finally, peripheral blood mononuclear cells transfer via EVs the chemokine receptor CCR5 to endothelial cells [112]. Endothelial precursors have been described to secrete EVs able to attenuate steroid-induced osteoblast apoptosis and autophagy, being able to upregulate glutathione peroxidase 4, system Xc^−^, and cysteine levels while reducing malondialdehyde and reactive oxygen species production [113].

**Figure 3:**
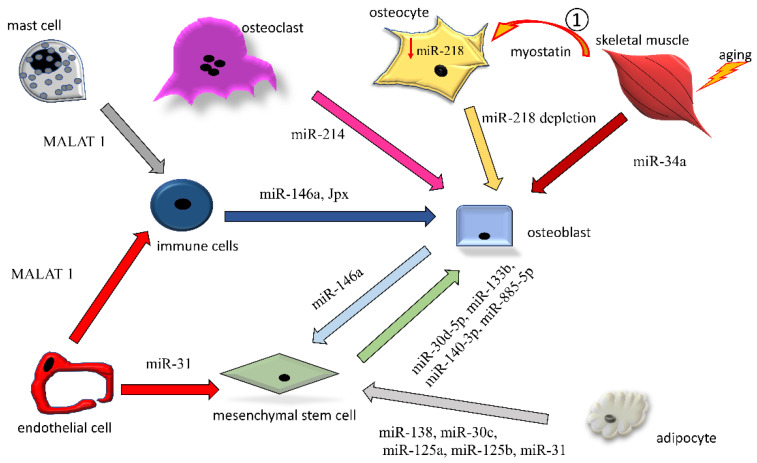
Intercellular exchanges of non-coding RNAs by means of EVs among bone-resident cells. Non-coding RNAs exerting osteopenic activity (increase in osteoclastogenesis or decrease in osteogenesis) are reported. Only ncRNAs directly found encompassed in EVs are reported in the figures. The arrows indicate the origin-to-destination direction between cells (donor to target cell). 1: soluble myostatin, released by aging muscle, affects osteocytes in a paracrine manner, reacting with the drop in endogenous miR-218a (↓miR-218a). The miR-218a-poor EVs released by the affected osteocytes are taken up by osteoblasts, contributing to the perturbation of osteogenesis.

Here, we discuss in more detail some examples of miRNA and lncRNA transfer by EVs among the bone cells, focusing on mechanisms detrimental to bone quality (Figure 3).

## 5. Epigenetic Detrimental Effects on Bone Metabolism by Means of EVs

### 5.1. Mesenchymal-Derived EVs

Human bone marrow stromal cells (hBMSCs) release EVs enriched in miRNAs, whose profile is different according to the metabolic status of the cells [114]. Among the identified miRNAs, Xu et al. characterized miR-885-5p. This miRNA was found inside EVs and its expression decreased during osteogenic differentiation of hBMSCs. Overexpression of miR-885-5p in hBMSCs impaired *wnt5a* and *runx2*, finally dampening their osteogenic potential.

Osteoblast-like cells MC3T3-E1 have been demonstrated to release EVs affecting mineralization of bone marrow stromal cell line ST2. These EVs were enriched in many miRNAs, among which miR-30d-5p, miR-133b, and miR-140-3p are reported [115]. A previous study already demonstrated that miR-30d-5p and miR-133b inhibit osteoblast differentiation targeting RUNX2, the master gene of osteoblast differentiation [116].

Different studies highlighted the association and contribution of miR-146a to the progression of different degenerative diseases, such as rheumatoid arthritis, systemic lupus erythematosus, and myelodysplastic syndrome, as well as osteoporosis, both in rodents and in patients [20,117]. Zhao et al. demonstrated that miR-146a supports RANKL expression in osteoblasts, enhancing osteoclast function and bone turnover, showing that miR-146a^−/−^ mice are protected from ovariectomy (OVX)-induced osteoporosis [117]. Cao and colleagues noted the TNFα-induced cell growth arrest of osteoblast-like MC3T3 cells by the upregulation of miR-146a [118]. Interestingly, immune cells are reported to secrete miR-146a-enriched sEVs [119]. Finally, Dong et al. reported that exosomes derived from the serum of systemic lupus erythematosus patients are enriched in miR-146a and induced the senescence of MSCs [120].

MSCs differentiate towards osteogenic or adipogenic precursors, and the osteoblast/adipocyte balance is tightly regulated at the level of gene transcription [121]. Adipogenesis is known to negatively affect osteogenesis and osteoblast function, and EVs from adipocytes participate in the inhibition of osteoblast differentiation [122]. In fact, MSCs treated with adipocytic EVs increased the transcriptional level of *PPARγ*, *leptin*, *CEBPα*, and *CEBPδ*, whereas they decreased *osteocalcin* and *osteopontin* levels [123]. These effects were ascribable to miR-138, miR-30c, miR-125a, miR-125b, and miR-31 miRNAs detectable in the adipocytic EVs, and in the MCSs upon incubation with adipocytic EVs, confirming a horizontal transfer of genetic material between these cells.

### 5.2. Immune Cell- and Osteoclast-Derived EVs

Ma et al. described that mononuclear cells and granulocytes release EVs shuttling the lncRNA JPX under some pathological circumstances in women [124]. Interestingly, JPX is known to be an activator of Xist. As previously reported, *Xist* is highly expressed in the serum and monocytes of patients affected with osteoporosis and induces inhibition of the osteogenic potential of bone marrow mesenchymal stem cells, inhibiting ALP, RUNX2, and osteocalcin expression [79].

Bone-resident mast cell proliferation, activation and degranulation are involved in the pathogenesis of osteoporosis [125]. The lncRNA profile of EVs from bone marrow-derived degranulated mast cells was analyzed by Liang and colleagues [126]. Interestingly, EVs were enriched in lncRNA MALAT-1, exerting a direct effect on macrophages/osteoclasts. Functional experiments on bone marrow macrophages revealed that, once transferred into target cells, MALAT-1 repressed miR-124, inducing overexpression of *mmp9*, *ctsk*, *acp5*, and *car2* genes and finally increasing osteoclastogenesis and bone resorption [127].

Sun and colleagues showed that osteoclasts secrete miR-214-enriched exosomes [128]. These exosomes are recognized via the EphrinA2/EphA2 axis and integrated by osteoblasts, inhibiting their activity. Exosomes from both OVX mice and osteoporotic patients contained high levels of miR-214 and ephrinA2 protein, suggesting that osteoclastic exosomes exert a noticeable inhibitory function on osteoblast activity in vivo. This mechanism was confirmed in OVX mice, since the prevention of exosome formation by downregulation of Rab27a increased osteoblast activity.

### 5.3. Endothelial-Derived EVs

Angiogenesis and bone remodeling are tightly related processes [129,130]. Both these processes can be heavily affected by senescence; in fact, both vascular and bone disfunction, as well as many other syndromes, can be alleviated by senolytic agents [131,132,133,134,135,136]. Weilner et al. demonstrated that miR-31 is present at elevated levels in the plasma of an elderly population [137]. Similarly, they reported serum miR-31 is elevated in the plasma of osteoporosis patients, therefore hypothesizing that miR-31 in the plasma of the elderly might play a role in the pathogenesis of age-related impaired bone formation. Looking at the source of miR-31, Weilner et al. identified senescent endothelial cells as producers, delivering miR-31 within EVs [137]. When miR-31 is taken up by mesenchymal stem cells, it inhibits osteogenic differentiation by counteracting its target *Frizzled-3* gene.

Endothelial cells can also affect osteoclastogenesis via EVs [127]. Endothelial progenitor cells secrete sEVs able to promote recruitment and differentiation of osteoclast precursors. These sEVs are enriched in lncRNA MALAT 1, exhibiting a pro-osteoclastogenic effect, as already discussed.

### 5.4. Muscle-Derived EVs

Bone homeostasis is tightly correlated with muscle performance and mass [138,139,140]. Along with the mechanic stimulation on the bone due to the kinetics of muscle contraction, muscle cells release specific cytokines, known as myokines, with anabolic/catabolic action on bone [141]. On this basis, osteosarcopenia, a term coined by Duque and colleagues, is a new emerging musculoskeletal syndrome [142]. In this context, EVs isolated from serum samples of elderly subjects showed a significant increase in a muscle-derived alpha-sarcoglycan positive EV subpopulation, compared to young controls [143]. Fulzele and colleagues showed that these EVs were also enriched in miR-34a. When they overexpressed miR-34a in mouse myoblast C2C12 cells by lentiviral vector infection and induced miR-34a human primary myotubes by hydrogen peroxide treatment (to simulate oxidative stress), the cells released EVs enriched in miR-34a, which in turn were able to abrogate *Sirtuin 1* (*sirt1*). *Sirt1* was reported also by other authors to be related to senescence in bone marrow stromal cells, decreasing osteogenic potential and bone deposition [144]. The authors concluded that their findings suggest that aged skeletal muscle is a potential source of circulating, senescence-associated EVs that may directly impact stem cell populations in tissues such as bone via their microRNA cargo.

Serum levels of muscle-derived cytokine myostatin increase with progressing age both in men and in women and are inversely associated with skeletal muscle mass [145,146]. Qin et al. showed that high levels of myostatin affect bone cells, especially osteocytes. In particular, Qui et al. described that the osteocytic cells Ocy454 and their sEVs contain miR-218 in basal condition [147]. Upon exposition of the myostatin, the Ocy454 cells increase SOST, DKK1, and RANKL production and decrease miR-218 expression. Interestingly, consequently to the cytoplasmatic drop in miR-218, the miR-218 content decreased in their sEVs. In addition, the same authors showed that osteoblast-like MC3T3 cells treated with miR-218-depleted osteocyte sEVs exhibited a stunted osteogenic differentiation. Finally, to confirm these data, Qin et al. demonstrated that exogenous overexpression of miR-218 in MC3T3 cells rescued the effect of myostatin-treated osteocyte sEVs, avoiding the increase in SOST and RANKL production, and the suppression of osteoblast differentiation and function. The authors concluded that, in addition to the effect on bone remodeling exerted through SOST and RANKL, the osteocytes can contribute to dampening osteogenesis by sEVs depleted in miRNA-218, adding one more EV-based mechanism contributing to altering the molecular asset of osteoblast function and osteogenesis.

A summary of all the miRNAs described above and their effects is reported in Table 1.

## 6. The Other Side of the Coin: EVs as a Potential Clinical Tool for Osteoporosis

In parallel with the studies elucidating the negative effect of EVs on bone metabolism and their involvement in the progression of skeletal diseases such as osteoporosis, many other reports highlighted a positive role of some EVs on bone metabolism, paving the way for their potential use in bone tissue engineering. This discrepancy can be explained by the difference in the molecular content of EVs, mirroring the status of the donor cells, as well as the asset of the target cells. Cui et al. showed that sEVs derived from mineralizing pre-osteoblast MC3T3-E1 cells can promote bone marrow stromal cell (ST2 cell) differentiation towards osteoblast [148]. Once taken up by ST2 cells, EVs induce a change in the miRNA profile of ST2, abrogating Axin1 expression while increasing β-catenin expression, overall leading to the activation of Wnt signaling and osteogenesis.

A study by Guo and colleagues demonstrated that sEVs from human synovial-derived mesenchymal stem cells (SMSCs) alleviate glucocorticoid-induced osteonecrosis of the femoral head (ONFH) in an experimental rat model [149]. Upon in vivo infusion, SMSC-EVs exert proliferative and antiapoptotic effects on bone marrow cells and improve bone mineral density and microarchitecture of femurs, increasing the number of osteogenic cells.

More recently, Liao and colleagues dissected the molecular mechanism of the ONFH progression in a rabbit model and the potential therapeutic use of mesenchymal stromal cell-derived EVs [150]. The authors showed that miR-122 is the most downregulated miRNA in ONFH. Interestingly, the *SPRY2* gene is a target for miR-122 and inhibits osteoblast differentiation via the RTK/Ras/MAPK signaling cascade suppression, and it has been reported to increase ONFH. Finally, EVs derived from bone marrow stromal cells overexpressing miR-122 were shown to attenuate ONFH development in vivo.

Endothelial cell-derived EVs were used as a preventive therapeutic tool in an experimental mouse model of steroid-induced osteoporosis [151]. The authors showed that dexamethasone induces ferroptosis in osteoblasts, suppressing glutathione peroxidase 4 (GPX4), system Xc^−^, and cysteine levels while upregulating malondialdehyde (MDA) and reactive oxygen species (ROS) production. EVs isolated by bone marrow-derived endothelial progenitor cells (BM-EPCs) injected in the tail vein of mice were able to mitigate the ferroptotic progression and increase bone parameters, such as bone volume, trabecular thickness, and structure model index. Although the authors did not investigate in detail the molecular players shuttled by EV-BM-EPCs, they attribute this feature to the miRNA and lncRNA content of EVs, since miR-17-92, miR-9, and miR-137, as well as lncRNA LINC00336, exhibit anti-ferroptotic activity.

## 7. Conclusions and Outlook

Knowledge about the function and the role of EV-based intercellular crosstalk adds one more piece in the intricate and crowded mechanism of the fine-tuned regulation of bone metabolism. It also contributes to understanding the molecular basis of the onset and progression of bone diseases. Indeed, EVs are an exciting tool with brilliant perspectives for clinical and theranostic development. These tiny structures and their molecular content could be used as biomarkers for diagnosis and monitoring of osteoporosis, as well as predictive and prognostic signatures.

Currently, some important limitations still dampen their use beyond the basic research. Major technical hurdles are still limiting the use of EVs as diagnostic tools [152]. The identification of the proper or best matrix or source (serum, plasma, urine) and the collection and the manipulation of the sample are still an unsolved aspect [153,154,155]. Furthermore, both the EVs and their molecular content can be modified by several patient-related variables, including the circadian rhythm and lifestyle habits such as physical activity [156,157,158]. Another important source of bias for EV-related RNA evaluation stems from the analytical platform, since results obtained from different technologies reported significant discrepancies in evaluating RNA levels, in conjunction with the applied normalization strategy [159,160].

However, mounting evidence supports the development of EVs as a diagnostic tool. EVs offer the invaluable possibility to obtain tissue-specific information with a non-invasive sampling procedure, the so-called liquid biopsy [161]. The stability of the EV molecular content is remarkable, since it can remain unaltered for many years when stored at controlled temperatures and is unchanged at extreme pH values and after several cycles of thawing/freezing [162,163]. The potential utility of EVs as a biomarker for bone diseases is further highlighted by the limitations of currently available diagnostic tools. The typical bone turnover markers (C-terminal type I collagen crosslinks (CTX), type I pro-collagen pro-peptide (PINP1), pyridinoline/deoxypyridinoline, parathyroid hormone (PTH), osteocalcin, bone alkaline phosphatase (BALP), and tartrate-resistant acid phosphatase 5b (TRAP5b)) are not completely accurate in evaluating bone formation and reabsorption, and dual-energy X-ray absorptiometry (DXA) provides information only about bone quality once important architectural alterations have occurred, often being irreversible [163]. Thus, we lack really efficient diagnostic tools to predict the risk or the evolution of metabolic bone diseases as well as the response to the treatments. Circulating EVs are promising biomarkers since they inform about epigenetic and transcriptomic modifications, making it possible to anticipate the alterations in the downstream signaling cascades and in classical protein markers.

EVs has been also proposed as a pharmacological platform for the treatment of musculoskeletal diseases [164]. EVs present attractive therapeutic potentiality due to a long half-life, high biocompatibility, and minimal or no adverse effects. Moreover, native (or engineered) EVs can be administrated to transport endogenous (or exogenous) molecules by their ability to target specific tissues. Regenerative medicine and targeted delivery of drugs benefit the most from the versatility of EVs. Degenerative and inflammatory disease, such as osteoporosis, can benefit from the therapeutic potential of EVs. EVs from mouse vascular endothelial cells are enriched in miR-155, exhibiting protective effects on bone in an ovariectomized (OVX) mice model of menopausal osteoporosis [165]. The pro-osteogenic potential of EVs released by mouse MSCs was specifically addressed to the bone by linking the murine MSC-derived EVs with alendronate [166]. These bone-targeting EVs were effective in protecting bone of OVX mice. In another study, murine osteoblast EVs were loaded with zoledronic acid, being able to suppress exacerbated bone resorption in mice [106]. However, some concerns (as reported for the diagnostic approaches) still limit EVs to becoming a concrete therapeutic system.

## Figures and Tables

**Table 1:** Intercellular exchange of miRNAs and their osteopenic effects.

Donor	Mediator	Target	Effect	References
Human bone marrow stromal cells	miR-885-5p	Human bone marrow stromal cells	Decrease in osteogenic ability suppressing *runx2* and *wnt5a*	[114]
Osteoblasts-like MC3T3 cells	miR-30d-5p, miR-133b, miR-140-3p	Bone marrow stromal cell line ST2	Inhibition of osteoblast differentiation by *runx2* suppression	[115]
Dendritic cells, MC3T3 cells, patients’ serum	miR-146a	Osteoblasts, bone marrow mesenchymal stem cells	Induction of cell growth arrest and senescence of osteogenic cells. Increase in RANKL/OPG ratio in osteoblasts.	[117,118,119,120]
Adipocytes	miR-138, miR-30c, miR-125a, miR-125b, miR-31	Mesenchymal stem cells	Suppression of osteocalcin and osteopontin levels	[123]
Mononuclear cells, granulocytes	lncRNA JPX	Bone marrow stromal cells	Reduction in *alpl*, *runx2*, *bglap*	[124]
Mast cells endothelial cells,	lncRNA MALAT 1	Macrophages, osteoclasts	Repression of miR-124, inducing the overexpression of *mmp9*, *ctsk*, *acp5*, and *car2*	[126,127]
Osteoclasts	miR-214	Osteoblasts	Inhibition of osteoblast function, sustaining of osteoporosis in OVX mouse model	[128]
Endothelial cells	miR-31	Mesenchymal stem cells	Inhibition of the osteogenic differentiation by suppression of *Frizzled-3*	[137]
Mouse myoblasts	miR-34a	Bone marrow stromal cells	Induction of *Sirtuin 1* reduction and senescence in bone marrow stromal cells	[143]
Myoblasts	Myostatin/miR-218	Osteocytic cells Ocy454	Decrease in osteocytic miR-218 leading to an increase in RANKL expression and decrease in SOST.	[147]
